# Impact of rapid molecular testing on clinical outcomes for methicillin-susceptible *Staphylococcus aureus* bacteremia

**DOI:** 10.1017/ash.2026.10749

**Published:** 2026-06-24

**Authors:** Jay Olivet, Matthew L. Brown, Megan Amerson-Brown, W. Seth Edwards, Robert A. Oster, Joshua Stripling

**Affiliations:** 1 UAB Hospital, Birmingham, USA; 2 UAB Medicine: UAB Health System, USA

## Abstract

**Background:**

Rapid molecular blood culture identification (BCID) enables earlier pathogen identification and targeted antibiotic therapy compared with traditional culture-based methods. Methicillin-susceptible *Staphylococcus aureus* (MSSA) bacteremia is optimally treated with anti-staphylococcal β-lactam antibiotics; however, delays in bacterial identification frequently result in prolonged empiric anti-methicillin-resistant *Staphylococcus aureus* (MRSA) therapy. Data evaluating the clinical impact of early MSSA identification remain limited.

**Methods:**

This retrospective, single-center, quasi-experimental program evaluation included adult patients with monomicrobial MSSA bacteremia. Patients were grouped based on identification by BCID or conventional culture-based methods. Automatic Infectious Diseases consultation was performed for all patients. The primary outcome was a desirability of outcome ranking incorporating treatment success, acute kidney injury (AKI), and inpatient mortality. Secondary outcomes included time-to-optimal therapy, duration of bacteremia, treatment success, and hospital length of stay.

**Results:**

A total of 300 patients were included (150 per cohort). BCID use was associated with earlier MSSA identification (0.8 vs 2.1 days; *P* < .001) and earlier initiation of targeted β-lactam therapy (1.9 vs 3.8 days; *P* < .001). The probability of a more desirable outcome with BCID was 58.5% (95% CI: 52.5% to 64.3%), and more individuals achieved the most desirable outcome (62.0% vs 46.0%; *P* = .005). AKI occurred less frequently in the BCID group (23.5% vs 44.2%; *P* < .001).

**Conclusions:**

Incorporation of rapid BCID into an established automatic Infectious Diseases consultation program improved outcomes in MSSA bacteremia by facilitating earlier β-lactam therapy and reducing nephrotoxicity. Antimicrobial stewardship programs should prioritize rapid diagnostics to optimize MSSA management.

## Graphical abstract

**Figure:**
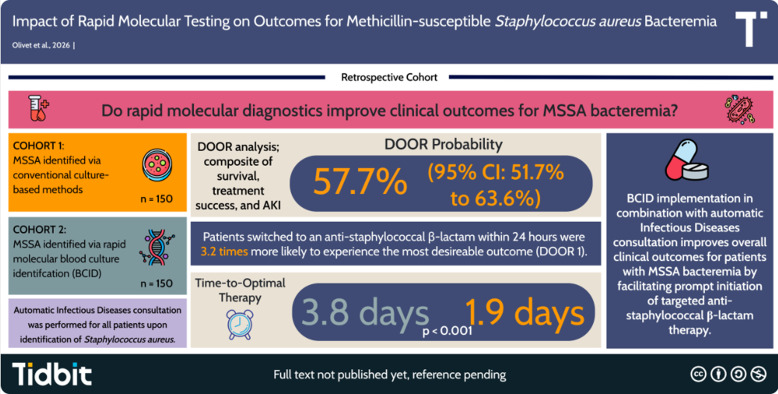

## Introduction

Methicillin-susceptible *Staphylococcus aureus* (MSSA) bacteremia is a severe bloodstream infection associated with high morbidity and mortality, particularly when treatment is delayed or suboptimal.^1,2^ Anti-staphylococcal β-lactam antibiotics, such as cefazolin or nafcillin, are preferred for definitive MSSA bacteremia therapy due to superior efficacy compared to vancomycin.^3,4^ However, in clinical practice, many patients receive empiric vancomycin therapy pending culture and susceptibility results, and delays in transition to targeted anti-staphylococcal β-lactam therapy have been associated with suboptimal clinical outcomes, including delayed clearance of bacteremia, higher rates of treatment failure, and nephrotoxicity.^2–4^

Rapid molecular diagnostics, such as blood culture identification (BCID) technology, offer the potential to expedite pathogen identification and allow for guidance of early antimicrobial optimization.^5^ These platforms use nucleic acid amplification techniques to identify microbial species and antimicrobial resistance genes directly from positive blood cultures, providing actionable information that can reduce the time to targeted therapy. Several studies have demonstrated that rapid molecular diagnostics in combination with antimicrobial stewardship intervention improve time-to-optimal therapy in bloodstream infections; however, data analyzing the impact of BCID for MSSA bacteremia remains limited.^6–9^ Given the well-established benefits of anti-staphylococcal β-lactam therapy for MSSA bacteremia, integrating BCID into routine clinical practice may enhance patient outcomes by reducing unnecessary vancomycin exposure and facilitating earlier transition to optimal therapy. This study aims to determine if identification of MSSA bacteremia via BCID compared to traditional culture-based methods results in meaningful clinical benefits.

## Methods

### Study design and population

This was a retrospective, single-center, quasi-experimental program evaluation study of adult patients with proven MSSA bacteremia identified by either conventional culture-based methods (preBCI group) or a BCID platform (BCID group) between January 2020 and August 2024. Patients were identified from a microbiology laboratory report and were included if they were ≥18 years of age, had laboratory-confirmed, monomicrobial MSSA bacteremia from any source who were treated empirically with an anti-MRSA agent and transitioned to a targeted anti-staphylococcal β-lactam at any point during the bacteremia treatment course. Patients who were transferred from an outside hospital, had polymicrobial infections, or died within 72 hours of treatment initiation were excluded from the study. In addition, patients in either group who underwent a patient-directed discharge without adequate outpatient therapy or had a delayed initiation of targeted anti-staphylococcal β-lactam due to antibiotic allergy were excluded. All data points were collected manually via chart review.

All blood cultures were incubated in a Bact/Alert Virtuo blood culture detection system (bioMérieux, Durham, NC). Upon bacterial growth, blood samples were Gram stained and plated using standard agar-based methods. Gram stain results were called to the provider. After implementation of BCID on February 1^st^, 2021, blood samples were loaded into the cobas eplex (Roche Diagnostics, Rotkreuz, Switzerland) panel corresponding with the Gram stain. Results for MSSA were reported in the electronic health record as “Rapid Molecular Identification: *Staphylococcus aureus*,” and a reference comment listed all potential targets that may be detected. Antimicrobial susceptibility testing was performed using a Microscan WalkAway Plus (Beckman Coulter, Brea California).

Dosing and monitoring of vancomycin was performed via institution protocol, which has been previously described.^10^ Upon identification of *Staphylococcus aureus* bacteremia, automatic Infectious Diseases consultation is performed at our institution. Infectious Diseases consult services review overnight automatic consultations the following morning.

### Outcomes

The primary outcome was a desirability of outcomes ranking (DOOR) analysis comparing overall patient outcomes between groups. The DOOR categories were based on treatment success (TS; defined as no ID physician directed change in therapy at any point during the treatment course due to inadequate response, no infection-related readmission within 90 days, and no recurrent infection within 90 days), acute kidney injury (AKI; defined as an increase in serum creatinine by ≥0.3 mg/dL within 48 hours or >1.5x baseline), and inpatient mortality. Treatment failure (TF) was defined by not meeting any of the three criteria for treatment success. The DOOR categories from most to least desirable were (1) survival with TS and no AKI; (2) survival with TS and AKI; (3) survival with TF and no AKI; (4) survival with TF and AKI; and (5) all-cause inpatient mortality.

Secondary outcomes included time-to-optimal therapy (defined as time from first positive blood culture collection to first dose of targeted anti-staphylococcal β-lactam), duration of bacteremia (defined as time from first positive blood culture collection to first negative blood culture collection), hospital length-of-stay (LOS), intensive care unit (ICU) LOS, incidence of infection-related readmission (defined as a readmission attributed to sequela of the index infection) within 90 days, incidence of recurrent MSSA infection within 90 days, incidence of TS, incidence of AKI, infection-related inpatient mortality, and all-cause inpatient mortality. This program evaluation study met institutional criteria for nonhuman subjects research.

### Statistical analysis

Descriptive statistics were used to describe categorical variables as instances and percentages and continuous variables as means and standard deviations. Proportions for categorical variables were compared between groups using the *χ* ^2^ test, or Fisher exact test if the assumptions for the *χ* ^2^ test were not satisfied. Means were compared using the 2-group t test. Distributions of continuous variables were examined using graphical techniques and statistical tests of normality. We concurrently ran the Wilcoxon rank-sum test to compare means between the groups since distributions of a few of the continuous variables deviated from a normal distribution; since results obtained from the two-group t-test and the Wilcoxon rank-sum test were similar, we present the results obtained from the two-group *t*-test for ease of interpretation and comparison with previous literature. The time-to-event analyses involved comparisons of group means, as described above, and did not include formal survival analyses. We have complete data on all patients; as such, we do not have any loss to follow-up, study withdrawals, or missing data on dates of therapy, so there are no censoring variables. In particular, time-to-optimal-therapy was computed as the number of days from the date of the first positive blood culture collection to the date of the first anti-staphylococcal β-lactam. Logistic regression analyses were used to obtain odds ratios and their corresponding 95% confidence intervals; these analyses were performed to obtain the likelihood of DOOR 1 (vs DOOR 2–5) for various intervals for time-to-optimal therapy. The point estimate and confidence interval for the DOOR probability were determined per Ong et al.^11^ Due to the exploratory, retrospective, and hypothesis-generating nature of this quasi-experimental study, no adjustments of *P*-values were conducted for statistical comparisons of the secondary outcomes. All tests of significance were 2-tailed, and a *P* value of <.05 was considered statistically significant. Statistical analyses were performed using SAS software (SAS Institute, Cary, North Carolina), version 9.4.

## Results

**Figure 1.**
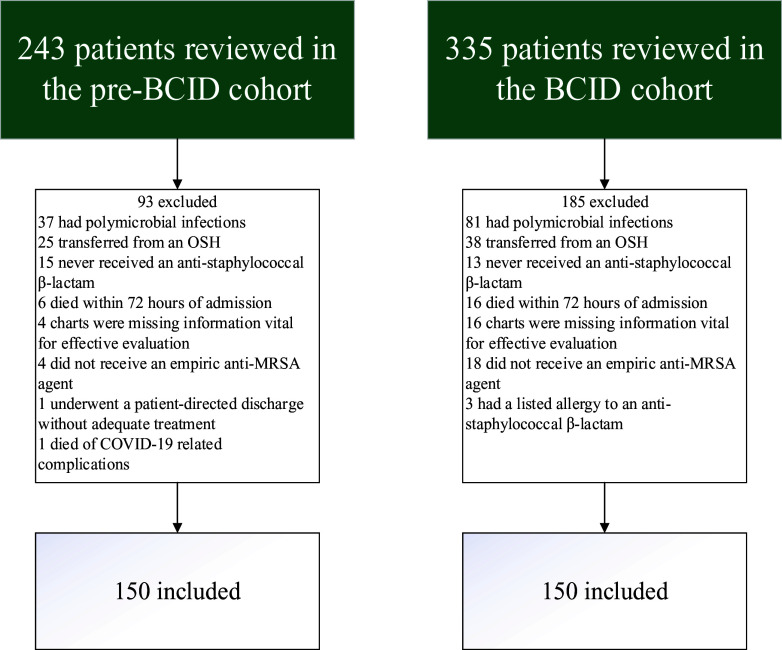
Study Population. Abbreviations: BCID, blood culture identification, OSH, outside hospital, MRSA, methicillin-resistant *Staphylococcus aureus*.

A total of 578 patients with MSSA bacteremia were evaluated for inclusion in the analysis. After exclusion, 300 patients were included with an equal number of patients in both the preBCID and BCID groups (Figure 1). Patient demographics and treatment characteristics were well matched among the groups and are outlined in Table 1 and Table 2, respectively. More patients in the preBCID group had COVID-19 at some point during the treatment course, but any deaths attributed to COVID-19 were excluded (n = 1). All other baseline characteristics, including percentage of patients requiring vasopressor support, mechanical ventilatory support, admission to ICU, and qPITT bacteremia score were similar between groups.^12^

**Table 1.** Baseline characteristics

Baseline characteristic	Pre-BCID (N = 150)	BCID (N = 150)	*P*-value
Age, years, mean ± SD	55.4 ± 16.5	58.5 ± 16.1	.096
Race			.333
White	91 (60.7)	106 (70.7)	
African American	52 (34.7)	38 (25.3)	
Hispanic	3 (2.0)	1 (0.7)	
Asian	2 (1.3)	2 (1.5)	
Unknown	2 (1.3)	3 (2.0)	
Male sex	106 (70.7)	91 (60.7)	.068
Weight, kg, mean ± SD	85.6 ± 25.9	84.2 ± 23.9	.611
BMI, kg/m^2^, mean ± SD	28.3 ± 8.0	28.3 ± 8.7	.994
Baseline eGFR, mL/min			.443
>60	101 (67.3)	109 (72.7)	
30–59	23 (15.3)	20 (13.3)	
<30	5 (3.3)	7 (4.7)	
iHD	21 (14.0)	13 (8.7)	
CRRT	0 (0)	1 (0.7)	
Chronic kidney disease	57 (38.0)	49 (32.7)	.763
Stage 1	4 (2.7)	6 (4.0)	
Stage 2	9 (6.0)	8 (5.3)	
Stage 3a	21 (14.0)	20 (13.3)	
Stage 3b	1 (0.7)	1 (0.7)	
Stage 4	22 (14.7)	14 (9.3)	
Diabetes	56 (37.3)	54 (36.0)	.811
Current IV drug use	18 (12.0)	12 (8.0)	.248
Immunocompromised	22 (14.7)	25 (16.7)	.809
Solid organ transplant	7 (4.7)	4 (2.7)	
Active cancer	11 (7.3)	14 (9.3)	
HIV with CD4 <200	0 (0)	1 (0.7)	
Hematopoietic stem cell transplant	1 (0.7)	1 (0.7)	
Immunosuppressing medications	3 (2.0)	5 (3.3)	
ECMO at treatment initiation	1 (0.7)	1 (0.7)	1.0
Mechanical ventilation at treatment initiation	18 (12.0)	12 (8.0)	.248
WBC at treatment initiation, mean ± SD	15.5 ± 7.2	16.9 ± 23.8	.491
Temperature at treatment initiation, °F, mean ± SD	100.7 ± 1.8	100.3 ± 1.9	.126
ICU at treatment initiation	51 (34.0)	63 (42.0)	.154
Vasopressor support at treatment initiation	23 (15.4)	34 (22.7)	.106
COVID during treatment	13 (8.7)	2 (1.3)	.004
Source of bacteremia			.745
Skin and skin structure	42 (28.0)	39 (26.0)	
Bone and joint	33 (22.0)	40 (26.7)	
Endovascular	24 (16.0)	22 (14.7)	
Catheter-associated	25 (16.7)	27 (18.0)	
Other^a^	19 (12.7)	16 (10.7)	
Unknown	7 (4.7)	6 (4.0)	
Septic emboli	36 (24.0)	29 (19.3)	.327
Altered mental status	47 (31.3)	60 (40.0)	.117
Penicillin allergy	24 (16.0)	22 (14.7)	.749
Complicated bacteremia	127 (84.7)	124 (82.7)	.639
qPITT score			.703
0	90 (60.0)	80 (53.3)	
1	37 (24.7)	42 (28.0)	
2	8 (5.3)	12 (8.0)	
3	13 (8.7)	15 (10.0)	
4	2 (1.3)	1 (0.7)	

Data are presented as No. (%) unless otherwise indicated.

BCID, blood culture identification; SD, standard deviation; BMI, body mass index; eGFR, estimated glomerular filtration rate; iHD, intermittent hemodialysis; CRRT, continuous renal replacement therapy; CKD, chronic kidney disease; HIV, human immunodeficiency virus; ECMO, extracorporeal membrane oxygenation; WBC, white blood cells; ICU, intensive care unit; qPITT, quick PITT bacteremia score.

a Other infection types include pulmonary (pre-BCID: n = 16, BCID: n = 14), intra-abdominal (pre-BCID: n = 3, BCID: n = 0), and urinary tract (pre-BCID: n = 0, BCID: n = 2).

**Table 2.** Treatment characteristics

Treatment characteristic	Pre-BCID (N = 150)	BCID (N = 150)	*P*-value
Vancomycin initial antibiotic	148 (98.7)	148 (98.7)	1.0
Vancomycin loading dose^a^	42 (28.4)	23 (15.6)	0.008
Days of initial antibiotic, days, mean ± SD	4.1 ± 1.6	2.3 ± 1.1	<.001
Doses on initial antibiotic, # doses, mean ± SD	6.3 ± 4.8	2.7 ± 1.5	<.001
Concomitant empiric gram-negative infection coverage at treatment initiation	140 (93.3)	139 (92.7)	.821
Initial antibiotic doses after identification of MSSA, doses, mean ± SD	2.6 ± 3.6	1.5 ± 1.5	<.001
Targeted combo therapy	7 (4.7)	12 (8.0)	.236
Definitive therapy			<.001
Cefazolin	117 (78.0)	140 (93.3)	
Nafcillin	33 (22.0)	10 (6.7)	
Total treatment duration, days, mean ± SD	34.6 ± 14.5	34.2 ± 14.8	.807
Inpatient treatment duration, days, mean ± SD	16.3 ± 10.7	17.3 ± 12.4	.490
Outpatient treatment duration, days, mean ± SD	18.3 ± 12.7	16.9 ± 13.3	.373
Source control achieved	82 (78.1)	59 (61.5)	.010
Duration of anti-staphylococcal β-lactam, days, mean ± SD	27.5 ± 14.7	27.7 ± 17.2	.931

Data are presented as No. (%) unless otherwise indicated.

BCID, blood culture identification; SD, standard deviation; MSSA, methicillin-susceptible *Staphylococcus aureus*.

a Pre-BCID: n = 148, BCID: n = 148.

The most common source of bacteremia was attributed to an acute bacterial skin and skin structure infection (ABSSSI) followed by intravenous catheter-associated source in both groups. About 12% of patients had endocarditis confirmed by echocardiogram (20 patients vs 17 patients in the preBCID and BCID groups, respectively). Most patients received a treatment duration of 4 or more weeks, which is indicative of complicated MSSA bacteremia (84.7% vs 82.7%, preBCID and BCID groups, respectively; *P* = .639). Vancomycin was the empiric agent used for 148 patients in each group; the remaining patients received daptomycin empirically. Patient management, including the antibiotic treatment course, was guided by formal Infectious Diseases consultation in each case.

**Figure 2.**
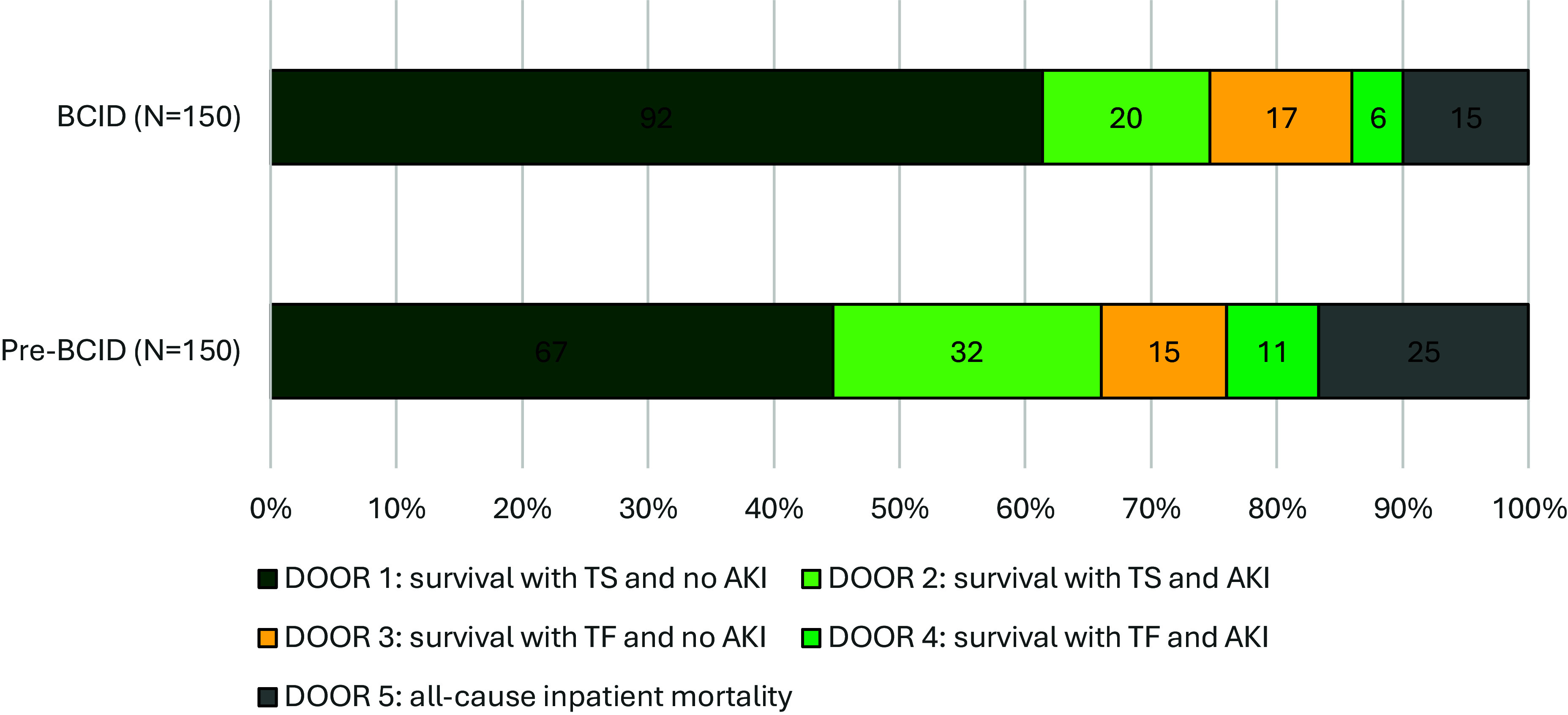
Desirability of Outcomes Ranking (DOOR) Analysis. Abbreviations: BCID, blood culture identification, TS, treatment success, AKI, acute kidney injury, TF, treatment failure.

The probability that a patient whose bacteremia was identified using BCID would have a more desirable outcome (based on the assigned DOOR category) than a patient whose bacteremia was identified using traditional culture-based methods was 58.5% (95% CI: 52.5% to 64.3%). In addition, more patients in the BCID group experienced the most desirable outcome (DOOR category 1) compared to patients in the preBCID group (61.3% vs 44.7%, respectively; *P* = .005, Figure 2). Secondary outcomes are displayed in Table 3. Incidence of AKI was lower in the BCID group compared to the preBCID group (23.5% vs 44.2%, respectively; *P* < .001). Hospital and ICU LOS, as well as infection-related and all-cause inpatient mortality, did not differ between groups.

**Table 3.** Secondary outcomes

Secondary outcome	Pre-BCID (N = 150)	BCID (N = 150)	*P*-value
Time-to-optimal therapy, days, mean ± SD	3.8 ± 1.4	1.9 ± 1.1	<.001
Duration of bacteremia, days, mean ± SD	4.1 ± 2.8	3.1 ± 1.8	<.001
Blood culture clearance <48 hr	32 (21.3)	57 (38.0)	.002
Blood culture clearance <72 hr	69 (46.0)	87 (58.0)	.038
Hospital LOS, days, mean ± SD	18.5 ± 13.6	23.5 ± 37.0	.120
Median (IQR)	13 (9-23)	13 (8–26)	
Escalation of care from floor to ICU	21 (14.0)	22 (14.7)	.869
ICU LOS, days, mean ± SD	10.4 ± 11.1	10.5 ± 8.9	.97
Median (IQR)^a^	10.4 ± 11.1	10.5 ± 8.9	
Time from collection to identification of MSSA, days, mean ± SD	2.1 ± 0.7	0.8 ± 0.4	<.001
All-cause inpatient mortality	25 (16.7)	15 (10.0)	.089
Infection-related inpatient mortality	21 (14.0)	13 (8.7)	.145
Infection-related readmission within 90 days^b^	26 (17.3)	14 (9.3)	.125
Reinfection within 90 days^b^	9 (6.9)	8 (5.9)	.741
Treatment success	99 (66.0)	112 (74.7)	.129
Developed AKI during treatment	57 (44.2)	32 (23.5)	<.001
AKI stage			.882
Stage 1	26 (45.6)	14 (43.8)	
Stage 2	10 (17.5)	7 (21.9)	
Stage 3	21 (36.8)	11 (34.4)	
AKI requiring RRT^c^	13 (22.4)	6 (19.4)	.737
CDI within 90 days	2 (1.3)	5 (3.3)	.448
VRE infection within 90 days	3 (2.0)	7 (4.7)	.198

Data are presented as no. (%) unless otherwise indicated.

BCID, blood culture identification; SDs, standard deviation; LOS, length of stay; IQR, interquartile range; MSSA, methicillin-susceptible *Staphylococcus aureus*; AKI, acute kidney injury; RRT, renal replacement therapy; CDI, *Clostridioides difficile* infection; VRE, vancomycin-resistant *Enterococcus*.

a Pre-BCID: n = 62, BCID: n = 81.

b Pre-BCID: n = 129, BCID: n = 134.

c Pre-BCID: n = 57, BCID: n = 32.

The BCID platform detected MSSA bacteremia earlier than traditional culture-based methods (0.8 vs 2.1 days; *P* < .001). Targeted anti-staphylococcal β-lactam therapy was started 1.9 days earlier in the BCID group compared to the preBCID group (1.9 vs 3.8 days; *P* < .001). Both factors led to significant reductions in anti-MRSA agent usage in the BCID group as described by the average duration and number of doses received (2.3 vs 4.1 days receiving an anti-MRSA agent; *P* < .001, and 2.7 vs 6.3 doses of an anti-MRSA agent; *P* < .001). Duration of bacteremia was also shorter in the BCID group (3.1 vs 4.1 days; *P* < .001).

**Figure 3.**
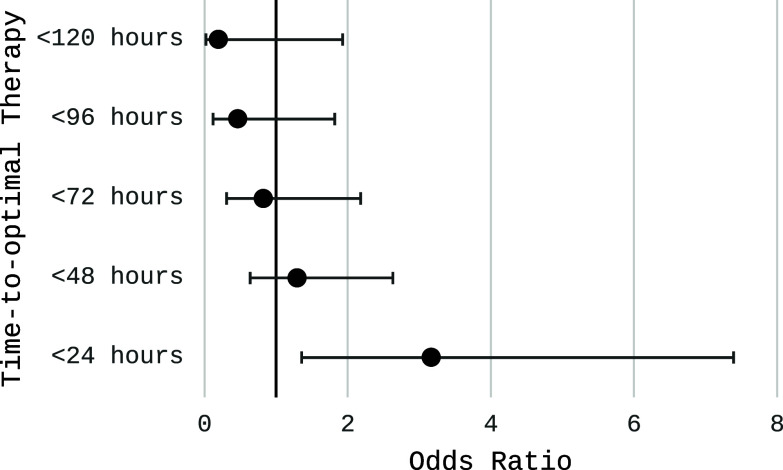
Likelihood of DOOR 1 by Time-to-Optimal Therapy in the BCID Cohort.

In a separate analysis, the likelihood of any patient achieving the most desirable outcome (DOOR category 1 vs DOOR category 2–5) was evaluated based on time-to-optimal therapy. When time-to-optimal therapy was less than or equal to 24 hours, the odds of a patient in the BCID cohort experiencing DOOR category 1 was 3.2 (95% CI: 1.4, 7.4; *P* = .008) as demonstrated in Figure 3. This analysis could not be performed for the preBCID group because zero patients were switched to optimal therapy in less than 24 hours. Other time points of 48, 72, 96, and 120 hours were evaluated but did not show statistically significant differences.

## Discussion

This study demonstrates that implementation of a BCID platform at an institution with an established automatic Infectious Diseases consultation program was associated with improved clinical outcomes for patients with MSSA bacteremia. These improvements coincided with earlier transitions to targeted anti-staphylococcal β-lactam therapy and reductions in vancomycin exposure, which may have contributed to the observed decrease in AKI rates.

Compared to traditional culture-based identification, BCID reduced the time to MSSA identification by 1.3 days and shortened time to optimal anti-staphylococcal β-lactam initiation by 1.9 days, which significantly reduced anti-MRSA agent use. Since vancomycin was the predominant empiric agent, these findings reflect how earlier organism identification and prompt initiation of targeted therapy may have mitigated downstream toxicity. The BCID group experienced significantly lower rates of AKI, despite similar baseline renal function. These findings are consistent with prior data associating prolonged vancomycin exposure with increased nephrotoxicity.^13,14^

Our findings are aligned with and expand upon prior studies evaluating the impact of BCID platforms on clinical outcomes. Nguyen *et al*. demonstrated that real-time polymerase chain reaction (PCR) detection of *mecA* significantly reduced vancomycin use and hospital LOS in patients with MSSA infections.^15^ Similarly, Drwiega *et al*. reported decreased vancomycin duration following rapid diagnostic implementation in a pediatric population with *Staphylococcus aureus* bacteremia.^16^ More broadly, multicenter evaluations, including Rule *et al*. and Chen *et al*., have shown that BCID panels accelerate time to effective therapy and can influence clinical decision-making across diverse populations.^17,18^ However, these studies largely focused on process outcomes rather than clinical outcomes. Our study adds that BCID not only expedites optimal therapy but also improves the overall quality of care for patients with MSSA bacteremia.

At our institution, identification of *Staphylococcus aureus* bacteremia triggers an automatic Infectious Diseases consultation. Thus, earlier identification of MSSA via BCID triggered earlier Infectious Diseases consultation and intervention. Prior studies have shown that early Infectious Diseases specialist involvement in *Staphylococcus aureus* bacteremia is associated with improved outcomes, including reduced mortality and recurrence.^19,20^

Though not adequately powered for these outcomes alone, the BCID group demonstrated a numerically higher treatment success rate (74.7% vs 66.0%) and a numerically lower all-cause mortality rate (10% vs 16.7%), which may be clinically meaningful. These outcomes are particularly notable given that numerically higher proportions of patients in the BCID group started treatment in the ICU (42.0% vs 34.0%), required vasopressor support (22.7% vs 15.4%), and achieved source control less frequently (61.5% vs 78.1%). Despite these markers of illness severity, the BCID cohort experienced a two-day reduction in median ICU length of stay (6 vs 8 days), which may offer substantial operational and financial benefits, as well as reducing the incidence of ICU-related mortality.^21^ Interestingly, the total duration of therapy and the duration on targeted anti-staphylococcal β-lactam therapy were similar between groups. This suggests that the observed improvement in outcomes in the BCID group was attributable to prompt initiation of optimal therapy rather than overall anti-staphylococcal β-lactam exposure. Time-to-optimal therapy comparisons further validate this point, as patients in the BCID group were 3.168 times more likely to experience the most desirable outcome when an anti-staphylococcal β-lactam was initiated within 24 hours of initial blood culture collection.

Several limitations warrant consideration. First, the retrospective, quasi-experimental design precludes definitive causal inference and introduces potential confounding. While groups were well matched at baseline, residual confounders may exist. Second, because therapeutic decision-making was guided by formal Infectious Diseases consultation, variable methods of recommendation communication and lack of around-the-clock automatic consultation service hours may have delayed timing of optimized therapy in some cases. Third, the generalizability of our findings may be limited to institutions with similar antimicrobial stewardship and rapid diagnostic infrastructure. Nafcillin was utilized as definitive therapy more in the preBCID group, but differences in AKI rates remain statistically significant when excluding patients who received nafcillin from the analysis. Due to institutional protocol changes during the study period, vancomycin loading doses were more common in the preBCID group; however, receipt of a vancomycin loading dose did not impact clinical outcomes or incidence of AKI, similar to previously reported findings.^22^

In summary, this study suggests that rapid BCID implementation in combination with automatic Infectious Diseases consultation improves overall clinical outcomes for patients with MSSA bacteremia by facilitating prompt initiation of targeted anti-staphylococcal β-lactam therapy. Our findings underscore the importance of expediting initiation of optimal therapy for MSSA bacteremia to improve clinical outcomes. Rapid diagnostics should be integrated into routine antimicrobial stewardship workflows to help optimize care for patients with bloodstream infections.

## Data Availability

Datasets can be made available upon request.
